# Gender bias in child custody judgments: Evidence from Chinese family court

**DOI:** 10.1371/journal.pone.0305479

**Published:** 2024-07-18

**Authors:** Xin Zhang, Shi Chen, Mengyuan Wang

**Affiliations:** 1 School of Law, Southwest University of Finance and Economics, Chengdu, China; 2 Policy and Law Division, Chengdu Normal University, Chengdu, China; Universita degli Studi di Messina, ITALY

## Abstract

Based on a quantitative analysis of a novel dataset comprising 10,093 publicly available judgments of adjudicated child custody disputes from the China Judgments Online website, this article identifies potential gender bias in Chinese family courts under certain conditions. Key findings include: 1. Mothers are generally more proactive in seeking custody and are awarded custody in the majority of cases compared to fathers. 2. Specifically, mothers have a significant advantage in cases involving daughters, while their advantage in cases involving sons is less pronounced. 3. In rural courts, the results are notably different: mothers are disadvantaged overall, fathers are particularly assertive in seeking custody of sons compared to daughters, and mothers are less likely than fathers to be awarded custody of sons. Building on existing literature, this study highlights potential judicial biases rooted in societal gender norms prevalent in rural areas. This raises questions about whether courts have achieved substantive gender equality and whether the legal principle of ’the best interests of the child’ is consistently upheld in every court decision. This study enhances the understanding of gender bias within China’s family court system by providing valuable insights for those interested in addressing gender inequality. It not only highlights specific challenges women face in custody cases but also calls for broader societal and policy changes to support women and combat gender discrimination in all its forms.

## 1. Introduction

The rising divorce rate has resulted in an unprecedented surge in custody dispute flooding China’s local courts. According to the Supreme People’s Court, in 2020, 4.339 million couples underwent divorce proceedings, with local courts handling 1.323 million divorce disputes, including 114,000 custody disputes. In contrast to jurisdictions such as the US, UK, and Australia, where shared parenting is emphasized [1], the most common post-divorce arrangement in China is the "sole" or "full custody" model, in which the child lives exclusively with one parent [2]. The noncustodial parent’s involvement in the child’s life is usually limited, either by personal choice or by interference from the custodial parent [3, 4]. China’s one-child policy, in effect from 1979 until its recent abolishment, limited families to a single child, increased the emotional and generational stakes attached to the only child [5], intensifying custody battles as losing meant to forfeit one’s only chance at parenthood [6]. The resulting "win or lose" dynamic in family courts often leaves one parent with full custody, exacerbating disputes and leading to extreme measures, such as child abduction [2, 7]. In this context, family court judges are faced with the complex task of determining the best living arrangements for the child.

While family courts around the world are gradually embracing the principle of "the best interests of the child," this standard requires that judges prioritize the welfare of the child to ensure decisions that are most beneficial to the child [8]. However, this seemingly gender-neutral legal principle can sometimes lead to unintended consequences, including possible gender bias in how courts allocate custody between fathers and mothers. Although empirical evidence suggests that when couples cannot negotiate a custody plan or achieve joint custody, maternal sole custody is the predominant arrangement in family courts in many countries, such as the US [9], Russia [10], Australia [11], the EU [12], Japan [13] and Hong Kong [14], with exceptions in certain religious and cultural contexts. Nevertheless, the debate about who wins custody cases, mothers or fathers, continues with some argue that mothers have an advantage [15–17], while others claim that custody arrangements discriminate against mothers [18–20].

Chinese family courts operate under the influence of two key elements: the gender policies within China’s legal framework and the societal norms and cultural attitudes regarding gender roles. China’s *Civil Law* and *Marriage Law* clearly stipulate that custody decisions should prioritize the welfare of minor children, requiring courts to remain impartial between parents. On the other hand, gender equality is a fundamental national policy in China and has been increasingly emphasized in recent years. Unlike in the Anglo-European framework, the concept of gender equality may be associated with a more individualistic approach to rights and equality, in China, it is often viewed through the lens of societal harmony and collective well-being. For instance, President Xi Jinping’s statement, "To make gender equality a reality, the protection of women’s rights must be elevated to the will of the nation," [21] reflects the Chinese government’s strong commitment to accelerating the achievement of substantive gender equality. The concept of substantive gender equality goes beyond formal or legal rights and emphasizes the need to provide women with tailored support and protection, while recognizing the differences in physiological and social roles between the sexes, in order to achieve true equality in society. Within this policy framework, family courts are charged with implementing the Communist Party of China’s central directive to safeguard the welfare of women and children, while also pursuing the policy goal of achieving substantive gender equality in individual cases [22]. Therefore, when dealing with family and marriage-related issues, it is not enough to simply adhere to the letter of the law. Instead, it calls for not only aiming at enhancing women’s legal rights and access to justice, but also foster a deep understanding and application of the principle of gender equality to ensure that each decision truly reflects this policy commitment.

While gender equality aims to reduce stereotypes and promote fairness between parents, the primary legal obligation of judges is to serve the best interests of the child. In China’s family courts, these two principles are generally compatible, with gender equality acting as a guideline to prevent discrimination rather than a rule that overrides the specific needs and best interests of the child. These tensions occur not because the principles themselves are in conflict, but because courts must consider factors such as economic stability, caregiving expectations, and the emotional and psychological needs of the child, which can sometimes lead to the de-emphasis of gender equality.

Equally important is the unique family culture in China, which can have a significant impact on the emergence and resolution of matrimonial and custody disputes. The traditional Chinese family culture, characterized by patriarchy, patrilineal systems, and a focus on paternal authority, is believed to establish a male-dominated ethical framework that relegates women to subordinate roles [23]. Studies focusing on rural communities [24–27] indicates that this framework may systematically disadvantage mothers in custody disputes. In addition, the cultural norms entrenched in Confucian and clan traditions, as analyzed in Chen [28] and Zhang’s [29] research, place a significant emphasis on the role of children, particularly sons, and underscore the expectation that they will financially support their parents in old age. The cultural preference for sons over daughters can reinforce gender power imbalances [26, 27], which challenge the equitable allocation of parental responsibilities and rights, as evidenced by social surveys indicating that daughters are more likely to live with mothers and sons with fathers after divorce [2, 7]. Anecdotal evidence and practices observed in some local courts suggest that there may be a male advantage in custody disputes involving sons [24, 26, 27, 30]. However, the lack of a comprehensive national study comparing urban and rural court practices in this area, highlights a gap in understanding these dynamics.

Given the ongoing global debate on gender bias in such decisions and the legal and societal mandates upheld by Chinese family courts, examining case outcomes in Chinese courts is essential. This study seeks to investigate several crucial questions regarding custody and divorce cases in Chinese courts. i) Which parent, mothers or fathers, prevails in custody disputes? ii) What are the outcomes of cases involving sons and daughters? iii) Are there differences in these outcomes between rural and urban courts? To answer these questions, we use a novel dataset covering custody cases heard by courts across China between 2014 and 2016, compiled from publicly available records on the *China Judgements Online* website.

Our research primarily focuses on identifying patterns in case outcomes. We use logistic regression models and decision tree techniques to explore potential relationships in the data. While we aim to provide some insight into why these patterns exist, our interpretations, based on existing literature rather than data results, are speculative rather than conclusive.

The significance of this research question stems from the fact existing empirical studies have yet to provide definitive answers to these questions. While specific quantitative analyses or local jurisdiction studies in China have produced conflicting results—some indicating advantages for fathers, while others argue for the predominant role of mothers (e.g., Chen and Zhang [24]; Zhao and Ding [25]; Michelson [26, 27]. Additionally, qualitative research has revealed the injustices and difficulties that women, particularly those in rural areas, encounter as they strive to assert their rights during divorce proceedings [31–35]. Thus, this study addresses a significant gap in the research landscape.

To our knowledge, this study is the first to leverage a nationwide dataset of court documents for a quantitative analysis of child custody decisions in Chinese family courts, enabling us to compare urban and rural jurisdictions and to investigate potential gender differences, an aspect rarely addressed in prior legal studies. By employing statistical methods to discern the factors influencing court decisions, our research extends beyond conventional methodologies. Consequently, it provides profound insights into the operations of Chinese family courts regarding custody and divorce, addressing a vital deficiency in existing legal and social science literature.

## 2. A review of factors influencing judicial outcomes in child custody cases

In this section, we review previous research that has examined factors that influence the outcomes of divorce and child custody disputes from three major perspectives. The first perspective focuses on differences between fathers and mothers in their willingness and ability to undertake parental responsibilities, and how these two factors affect the outcomes of disputes. Mothers are more likely to take the legal initiative in seeking custody [36–38], largely due to societal expectations of them as the natural caregivers. However, financial constraints may limit a mother’s ability to pursue legal action. [39–42].

In the fewer instances where fathers proactively seek custody, they often have greater bargaining power due to their higher income and earning capacity [43, 44]. In addition, fathers may use threats, violence, or other coercive methods to pressure the opposing party during negotiations [39, 40]. This issue is particularly significant in Chinese courts, where male litigants often exhibit uncooperative behavior, and sometimes resort to violence. These tactics intimidate opponents and complicates the legal process, affecting the overall fairness of the proceedings [3, 26, 33, 34].

The second perspective examines how social norms and practices regarding gender roles influence the outcomes of cases, resulting in a bias in favor of one parent over the other, even when the laws appear to be gender neutral. For example, when judges apply the "tender years doctrine," mothers are often favored due to traditional beliefs that they are inherently better suited to care for young children, based on presumed maternal instincts and abilities. However, the presumption of primary caregiving, grounded in developmental research emphasizing the importance of continuity of care, requires judges to consider the specific circumstances of each case. This presumption generally results in outcomes favoring mothers [15]. Nonetheless, it can sometimes favor fathers, either due to their direct caregiving role or the involvement of paternal grandparents in the child’s care. The adoption of joint custody arrangements may inadvertently favor fathers [18]. Although intended to provide more balanced parenting time and address inequities in caregiving roles during marriage, these arrangements may unintentionally favor fathers in the quest to equalize parental involvement.

In Chinese courts, the legal principle of "maintaining the status quo of current living conditions" serves to safeguard children’s emotional attachments and ensure the stability of their existing living environments. However, this principle can complicate custody battles for rural women, who typically live with their husbands’ families and leave their natal homes upon divorce [26]. Consequently, male litigants are more likely to retain physical custody of the children [27]. Thus, courts might favor keeping children in the paternal home, believing it offers greater stability and aligns with the child’s current living situation, which could place mothers at a disadvantage in custody disputes.

Judges’ emphasis on a parent’s “ability to support the child” makes women less likely to receive favorable outcomes because they are more likely to face financial challenges compared to men, and they are often at a disadvantage in court-determined divorce property settlements [45]. The patrilineal system prevalent in rural areas further contributes to their economic vulnerability [46]. This manifests in three main ways: First, due to scarce employment opportunities and lower incomes in rural areas, women often rely financially on their husbands. The dependence weakens their bargaining power [47]. Second, the patrilineal system often prioritize men’s rights over women’s rights, especially in property and inheritance matters. As a result, women have limited access to land and other assets [48]. Third, the lack of formal documentation of land rights and marital property makes it difficult for women to assert ownership or secure a fair share after divorce [45].

"Son preference" is a well-documented phenomenon across various cultures [26, 27, 41, 42]. However, the underlying reasons for this trend differ significantly. In developed societies such as the U.S., studies emphasize that fathers are more likely to assertively seek custody of their sons mainly because fathers believe they are better equipped or suited to raise sons [49, 50]. Conversely, in China and similar cultures, research indicates that both parents may prefer sons. This preference may stem from the perception that sons provide greater economic support to aging parents than daughters who marry out [28, 29]. Furthermore, in patriarchal societies, sons are more valued for the maintenance of the paternal line or the inheritance of family wealth [51, 52].

The third perspective underscores judges’ gender stereotypes or personal beliefs about parenting and child development [42, 53, 54]. For example, when judges view mother as the more suitable caregivers [45], fathers sometimes have to make greater efforts in court proceedings to secure custody [42, 54]. Conversely, mothers may be held to a significantly higher standard in parenting evaluations because of higher societal expectations of their parenting role [17, 19]. Empirical studies also indicate that judges in Chinese courts tend to award the custody of children to parents of the same gender [24], and in particularly, to award custody of daughters to their mothers, [55–57], echoing the prevailing social view that mothers are better suited to care for daughters. Fathers are granted custody of daughters only for their exceptional involvement or advantages in parenting [57].

Some studies criticize judges for making decisions with clear gender bias, such as awarding custody to fathers in mediations where the female party is persuaded to relinquish custody of a son [35], under the notion that "the male family line must be preserved" [26]. In some cases, judges have awarded custody to fathers despite evidence of domestic violence or clear indications that they were legally or morally unfit to assume parental responsibility [26, 27, 35]. Instead of maintaining neutrality between the parties, judges deferred to or conformed to patriarchal gender norms in these cases.

The uneven playing field for women and men in Chinese courtrooms has been interpreted for institutional and political reasons [24, 26, 48]. Judicial decisions in favor of fathers, who are often impulsive and disruptive, are more in line with judge’s professional interests [22, 47], as this can avoid antagonizing male litigants and the potential risks they pose [34]. Moreover, judges are often encouraged to seek mediated solutions that are generally acceptable to all parties rather than resolving disputes through litigation [27, 34]. In this process, rights-based claims are often diminished to interest-based bargaining, where women’s legal rights become negotiable commodities [27, 32, 34].

## 3. Legal framework for resolving custody allocation disputes in China

Under China’s Civil Law, spouses have equal rights and responsibilities with respect to the obligation to raise, supervise, educate, and protect their offspring, regardless of their marital status. However, the most common post-divorce custody arrangement in is for the child to live solely with one parent, despite the law recognizes alternate custody and other shared custody arrangements. Noncustodial parents’ rights to remain involved in their children’s lives go unfulfilled because the other parent often deliberately blocks them [3, 4]. When divorcing parents have difficulty agreeing on child custody, they turn to the courts to determine custody arrangements. This typically involves decisions about physical custody—where the child will live after the divorce and which parent will have primary responsibility for the child’s day-to-day care.

Child custody claims are handled as part of divorce proceedings, sometimes along with the claims for division of marital assets and child support orders. These cases usually conclude in one of three ways: (i) voluntary dismissal by the involved parties; (ii) settlement achieved through court-mediated negotiations; (iii) a final judgment issued by the court. According to data from the Supreme People’s Court [58], voluntary dismissals and mediated settlements represent roughly 70% of all cases. The remaining cases, which require a formal judicial decision, tend to be the most complex and hotly contested.

Most parents hire lawyers because cases brought to court are usually highly contentious and difficult to resolve privately. While family court judges generally have law degrees, they do not always have specialized training in family law. Furthermore, there is no comprehensive public data on the gender distribution of these judges. The court investigation process remains predominantly judge-centered. According to the "Opinions on Further Deepening the Reform of Family Trial Methods and Working Mechanisms" issued by the Supreme People’s Court in 2018, courts may appoint family investigators to conduct investigations when necessary. However, until recently, this practice was quite rare and mainly limited to pilot programs in certain regions, without being fully institutionalized nationwide.

When judges need to consider a child’s wishes, they may use videotapes to determine the child’s wishes or, less commonly, interview the child in more informal settings outside the courtroom. Inviting the child to appear in court is up to the judge. Notably, the rate of children appearing in court is low, often because they are unwilling or because their parents advise or pressure them not to appear.

Prior to 2021, child custody in China was primarily governed by Article 36 of the *Marriage Law*. If the parties cannot agree on custody through negotiation, the court will make a decision based on the principle of "protection of the interests of minors" (similar to "best interests of the child" in Western jurisdictions), except in cases involving breast-feeding infants, where the child is generally awarded to the mother. The *Opinions on Handling Child Support Issues in Divorce Cases* (hereinafter referred to as the *Opinions*) [59], issued by the SPC in 1993, has laid down detailed guidelines. Although it was replaced by the Civil Law and its judicial interpretation in 2021, the core content remained largely the same. The legal guidelines suggest that judges should consider the following five legal principles when determining custody of children over the age of two: First, judges are advised to prioritize awarding custody to a parent who has lost the ability to conceive or has no other children. This guideline, rooted in family planning policies that led to many women being sterilized after having one child, aims to protect women’s interests. Second, judges should consider maintaining the child’s current living situation by granting custody to the parent who has been the primary caregiver. This minimizes disruption in the child’s life. Third, if either parent has serious health or other issues that may detrimental to the child’s well-being, judges are advised to disqualify them from custody. Fourth, the principle of intergenerational support suggests that if a child’s grandparents have been the primary caregivers, it may be preferable to grant custody to the grandparents’ child. Finally, for children aged eight or older, the court considers their expressed preferences. In cases involving two or more children from the same marriage, it is customary for judges to take an egalitarian approach by dividing the children between both parents and ensuring that each parent retains an active role in their children’s lives [24, 27].

Given the above, we can summarize that the legal framework for child custody disputes in China is gender neutral. As it contains certain provisions of special protection for women’s rights, it conveys a sense of substantive gender equality. However, the administration of these legal principles is unknown. Given that many of them are advisory rather than mandatory, a variety of factors may influence the judicial process and its decisions, including judges’ personal beliefs and potential biases.

## 4. Data and methods

We constructed a dataset of 10,093 initial legal decisions made by courts between 2014 and 2016 to determine the award of custody of a single minor child. This selection was sourced from *China Judgements Online*, an official website mandated by the Supreme People’s Court of China since 2013, which requires courts at all levels to publish their decisions. The specific time period was chosen based on a significant policy shift by the Supreme Court in October 2016, which mandated that divorce-related decisions and decisions involving the custody and child support be excluded from public access to protect the privacy of the parties involved.

We conducted a thorough online search using the keywords “divorce litigation” and “child custody disputes”. As of November 2021, our search yielded 16,175 preliminary court decisions regarding the child custody, 14,312 of which involving a single child. We then removed cases where custody was voluntarily relinquished by one party, cases where there was no real dispute, or cases with no information regarding the child’s gender, court details, or the identities of the parties involved.

Despite potential concerns about the sample bias because the criteria used by the courts to decide which judgments to upload and when to upload them remains unknown, several factors suggest that this bias is likely to be minimal in this study. First, previous studies indicate that the volume of cases positively correlates the frequency of public disclosures [37]. Divorce and custody cases courts have a higher rate of public disclosure than other types of cases because they are perceived to be less politically sensitive. As a result, courts have greater autonomy in publishing these judgments. Second, the sample is large and representative. This extensive sample of decisions comes from 2,160 grassroots courts, representing 69% of all such courts nationwide. This broad representation enhances the credibility and robustness of our data and allows for comprehensive analysis across a wide range of court settings in China.

Although the potential impact of case selection effects [60], particularly in privately settled cases, may alter the extent of judicial involvement in divorce and custody decisions, research by Wang [61], Li [34], and He [33] has shown that gender bias is more pronounced in cases resolved through negotiation and mediation. These findings reinforce the relevance and importance of our research.

We chose not to analyze the detailed narratives of the judgments because judges often avoid extensive explanations but rather use ambiguous terms or make generic statements that obscure their actual reasoning and opinions [62]. Instead, we focused on case outcomes, case information, and decision-making approaches to examine the factors that influence case outcomes without relying on potentially unclear judicial narratives.

### Case outcomes

We looked at whether custody of the child was awarded to the father or the mother.

### Case information

We identified the year of the case, the sex of the child, and whether the child was born in or out of wedlock.

We also scrutinized the parental roles as plaintiffs or defendants and their expressed attitudes towards custody in court documents, ranging from firm custody claims to ambiguity or outright renunciation. Attitudes such as "abide by the court’s decision" or "respect the child’s or other party’s wishes" were considered ambiguous.

The "assertive plaintiff" variable highlighted cases where a clear, firm stance on custody rights was articulated, accounting for 3,762 cases (37.27%).

### Judicial decision-making approaches

We examined whether the case entailed matrimonial property settlement and if it involved significant amounts, judged by litigation costs exceeding the standard threshold of 300 RMB, indicating matrimonial assets above 200,000 RMB, termed as “substantial matrimonial property."

We recorded whether the parties requested the court to make a child support order.

We noted the geographical location of the court, whether urban or rural.

We recorded the procedure adopted by the court, whether simplified or regular.

The location of the court reflects backgrounds of the litigants and the socio-cultural context of their dispute, as litigants must file their cases in local courts where they reside or are registered. In China grassroots courts are located in counties, county-level cities, autonomous counties and municipalities. We followed the standard definitions of urbanization in determining the location of the courts: those in municipalities and county-level cities were classified as urban, accounting for 68% of all cases studied, while those in counties and autonomous counties were classified as rural, accounting for 32% of the custody disputes.

Another variable used to measure judicial decision-making is the procedure adopted by the court: simplified or regular. According to China’s Civil Procedure Law, courts can choose between simplified and regular procedures to balance judicial efficiency and fairness through reasonable procedural arrangements, ensuring that different types of civil cases are appropriately handled. Simplified procedures, conducted by a single judge, are typically used for simple civil cases with clear facts, defined rights and obligations, and minor disputes. For example, cases in which defendants are unresponsive or use passive legal defenses, such as "no plea" or "no evidence" strategies, often fall within the scope of simplified procedures, indicating minimal active participation by the defendant. In addition, cases with established custody criteria, such as those involving breast-feeding infants, may also follow a simplified procedure. In contrast, regular procedures are conducted by a collegial panel, usually consisting of three or more judges, and are appropriate for more complex or contentious cases that may require in-depth hearings and investigations.

The choice of procedure reflects the complexity of the case, which illustrates the effort and contention between the parents as plaintiffs and defendants in the custody battle, and the judge’s strategy for handling the case. In highly contentious cases, judges may perceive greater risks and thus form a collegial panel to mitigate these risks, as noted in [63]. By engaging in collective decision-making, judges participate equally in the trial, deliberation, and sentencing process, allowing them to gather diverse perspectives and ensure more comprehensive and fair decisions by minimizing potential errors and biases. This approach not only reduces the risks faced by a single judge in complex cases, but also more effectively manages extreme behavior between parties, such as intense confrontation, malicious accusations, or even threats of violence.

We also recorded the specific legal provisions that judges referred to in their decisions. Referring to the *Opinions* indicates that judges were seeking more explicit legal principles and standards rather than relying solely on their discretion.

We observed a recurring trend of mothers being awarded custody and often initiating these litigations, as shown in Table 1. Interestingly, a slightly higher number of cases involve boys than girls, and about 12.04% of the disputes involve children born out of wedlock. 32.06% of cases are filed in rural courts, reflecting the more conservative attitude of urban dwellers towards divorce [64] or a greater reluctance to use the courts to resolve family issues.

**Table 1:** Data description.

Item	Frequency	Percentage
**Custody award**		
Mother	5835	57.81%
Father	4258	42.19%
**Plaintiff**		
Mother	7295	72.28%
Father	2798	27.72%
**Assertive plaintiff**		
(Did the plaintiff make a decisive claim for custody rights?)		
No	6331	62.73%
Yes	3762	37.27%
**Child gender**		
Boy	5528	54.77%
Girl	4565	45.23%
**Court district**		
Rural	3236	32.06%
Urban	6857	67.94%
**Year**		
2014	3288	32.58%
2015	3798	37.63%
2016	3007	29.79%
**Child in wedlock**		
(Are the child born in wedlock?)		
Yes	8879	87.97%
No	1214	12.04%
**Child support order**		
(Do the parties request a child support order from the court?)		
Yes	7538	74.69%
No	2555	25.31%
**Procedure**		
(Is the case adjudicated by a panel of judges or a single judge?)		
Regular	4832	47.87%
Simplified	5261	52.13%
**Claims with matrimonial asset settlement**		
(Do the parties request the court to settle matrimonial assets?)		
No	6951	68.87%
Yes	3142	31.13%
**Settlement involving substantial matrimonial assets**		
(Does the value of the matrimonial property to be divided exceed 200,000 RMB?)		
No	8982	88.99%
Yes	1111	11.01%
**Child age (<2)_ Article**		
(Does the court refer to legal provisions favoring the award of custody to mothers of children under two years?)		
No	9799	97.09%
Yes	294	2.91%
**Intergenerational Supports _ Article**		
(Does the court consider awarding custody to the parent whose grandparents have primarily cared for the child?)		
No	9930	98.39%
Yes	163	1.61%
**Child opinion_ Article**		
(Does the court take into account the child’s preferences when both parents are equally capable of providing care?)		
No	9767	96.77%
Yes	326	3.23%
**Exclude unfit parent_ article**		
(Is disqualification of a parent for serious health issues or unfitness considered?)		
No	9710	96.21%
Yes	383	3.79%
**Balancing parents’ interests_ article**		
(Does the court give preference to childless or infertile parents when deciding custody?)		
No	9686	95.97%
Yes	407	4.03%
**Maintain status quo_ article**		
(Does the court favor maintaining a child’s current living situation to ensure stability?)		
No	9553	94.65%
Yes	540	5.35%

Almost half of these cases follow regular procedures. This underscores the high level of contention between the parties in child custody disputes. In addition, a substantial majority of cases (74.69%) involve claims for child support order. Notably, a significant portion of disputes do not involve marital property issues (68.87%), indicating that financial disagreements may have a limited impact on case outcomes. However, the remaining 31.13% of cases, particularly those involving significant property settlements (11.01%), present more challenges, signaling increased animosity between the divorcing couples.

We find that the decisions are often made without applying specific legal provisions. This could be because judges deliberately avoid doing so, or because these cases do not meet the conditions for applying these legal provisions. As a result, a significant portion of decisions are based on judges’ discretion. Therefore, the personal beliefs, attitudes, and opinions of judges have a significant impact on the results.

We adopt several approaches to explore the effects of various factors on the case outcome. To assess the relationship between a single factor and case outcome, we construct contingency tables (and corresponding percentages) and determine the statistical significance using chi-squared tests.

Furthermore, a multivariate linear logistic regression is adopted to assess the concurrent effects of various factors on the case outcome. Case outcome (custody award to mother or father) is the response variable, and all factors are included as independent variables. To further address the interactive effect between court district and child gender as well as between plaintiff claims and plaintiff gender, corresponding interaction terms are also included in the model. (see Eq (1)).


logit[P(CustodyAward=Father)]=Year+ChildSupportOrder+Childinwedlock+Procedure+Involvingsubstantialmatrimonialassetssettlement+Claimswithmatrimonialassetsettlement+Assertiveplaintiff+plaintiff+Courtdistrict+Childgender+Assertiveplaintiff*plaintiff+Courtdistrict*Childgender (1)

To better understand the nuanced gender-based outcomes in custody disputes across urban and rural settings, a GUIDE decision tree was employed [65]. Unlike logistic regression, which assigns a fixed form of relation between each variable and outcome, this method allows for the identification of distinct subgroups whose outcomes differ significantly from the general population. GUIDE models different types of data and recursively selects factors to split the data in an unbiased manner. Final results from GUIDE display all cases in several mutually exclusive subgroups defined by various factors, which provides us an alternative way of understanding which factors may impact the case outcome most. This allows for a more vivid and a visual exploration of how different factors influence case resolutions in different legal landscapes.

To further exam the case outcome on boys’ custody in rural courts, we construct contingency tables to correlate the case outcome and other factors. The statistical significance is determined using chi-squared tests.

Overall, we considered p value < 0.05 to be statistically significant.

## 5. Results and discussions

On average across the country, mothers are granted custody in 57.81% of cases, whereas fathers are granted custody in 42.19% of cases. Mothers often take a more proactive legal approach compared to fathers, in both urban and rural settings, and they typically acting as plaintiffs in 72.28% of cases. The pattern not only reflects the mothers’ predominant role in child-rearing but also indicates increasing legal awareness among women and their heightened use of legal mechanisms to assert their rights.

The reluctance of fathers to pursue custody may be due to a number of factors, including a reluctance to engage with the legal system. Although fathers are less likely to initiate legal action, when they do step forward to assert their claims as plaintiffs, they present stronger cases. This strategic approach may result in a greater likelihood of judicial support for fathers than when mothers initiate proceedings, as shown in Table 2.

**Table 2:** Parameter estimates of the multivariate logistic regression model on case outcome. Positive estimates indicate that it is more likely to award the child to father.

	Estimate (SE)	Odds Ratio (95% CI)	P-value
**Simplified procedure**	0.10 (0.042)	1.14 (1.05, 1.242)	0.0156 *
**(year)2015**	0.01(0.050)	0.99 (0.897, 1.093)	0.8483
**(year)2016**	0.08 (0.054)	1.09 (0.98, 1.209)	0.1153
**Child support order**	-0.13 (0.049)	0.88 (0.799, 0.967)	0.0083**
**Child in wedlock**	0.05 (0.065)	1.05 (0.924, 1.193)	0.4554
**Father as plaintiff**	0.28 (0.058)	1.32 (1.177, 1.479)	<0.0001***
**Assertive plaintiff**	−0.87 (0.055)	0.42 (0.376, 0.465)	<0.0001***
interaction: Assertive plaintiff * Father as plaintiff	1.13 (0.096)	3.09 (2.562, 3.737)	<0.0001***
**Child sex: girl**	−0.61 (0.074)	0.55 (0.472, 0.63)	<0.0001***
**Urban district**	−0.69 (0.060)	0.5 (0.444, 0.562)	<0.0001***
Interaction: urban district * girl	0.22 (0.090)	1.25 (1.046, 1.489)	0.0141*
**Involving a substantial matrimonial property settlement**	−0.31 (0.075)	0.73 (0.632, 0.846)	<0.0001***
**Claims with matrimonial property settlement**	−0.05 (0.048)	0.95 (0.865, 1.045)	0.2951

SE: Standard Errors. For variable “Simplified Procedure”, the estimates, odds ratio and p value are for the comparison of Simplified procedure vs Regular procedure; for variable “Child Sex: Girl”, the estimates, odds ratio and p value are for the comparison of Girl vs Boy; for variable “Father as plaintiff”, the estimates, odds ratio and p value are for the comparison of Father vs Mother; for variable “Urban district”, the estimates, odds ratio and p value are for the comparison of Urban vs Rural; For other independent variables, the estimates, odds ratio and p value are for the comparison of Yes vs No. The p value of the logistic regression model against the null model is < 0.0001.

The results of the logistic regression show significant variations in case outcomes based on the sex of the child and the court location. As shown in Table 3, mothers are 28.14% more likely to be awarded custody in cases involving girls compared to fathers, whereas this advantage drops to 5.28% in cases involving boys. In urban courts, mothers are 25.66% more likely to be awarded custody. In rural courts, fathers are 5.62% more likely to be granted custody than mothers.

**Table 3:** Case outcome by district and child gender.

Court district	Rural		Urban		Overall	
Custody award	Mother	Father	Mother	Father	Mother	Father
**Boy**	740	1086	2170	1532	2910	2618
	(40.53%)	(59.47%)	(58.62%)	(41.38%)	(52.64%)	(47.36%)
**Girl**	787	623	2138	1017	2925	1640
	(55.82%)	(44.18%)	(67.77%)	(32.23%)	(64.07%)	(35.93%)
**P-value**	< .00001		< .00001		< .00001	

When considering both the gender of the child and the location of the court, the gendered outcomes become more pronounced. In urban courts, mothers are more likely to be awarded custody of girls and have a slight advantage in cases involving boys. However, in rural courts, fathers lag behind mothers in cases involving girls but hold an advantage in cases involving boys, reversing the general trend.

When examining the relationship between procedural types—simplified versus regular—and custody outcomes, clear patterns emerge. Fathers are more likely to be awarded custody under simplified procedures, indicating that these decisions are typically made in less contentious cases with lower decision-making risks. Conversely, mothers are more likely to be awarded custody in cases handled through regular procedures, suggesting that decisions in favor of mothers tend to involve more complex and contentious situations. One possible explanation is that judges in these cases anticipate greater scrutiny or backlash, particularly from fathers, and therefore establish a collegial panel to mitigate potential decision-making risks.

Additionally, the logistic regression result suggests that custody of the child is awarded to the mother when the cases involve the division of substantial marital assets and when there is also a concurrent claim for child support order. This indicates that when there is no financial uncertainty regarding the child’s upbringing, judges are more likely to view the mother’s role in the daily care and upbringing as being in the best interest of the child.

**Fig 1:**
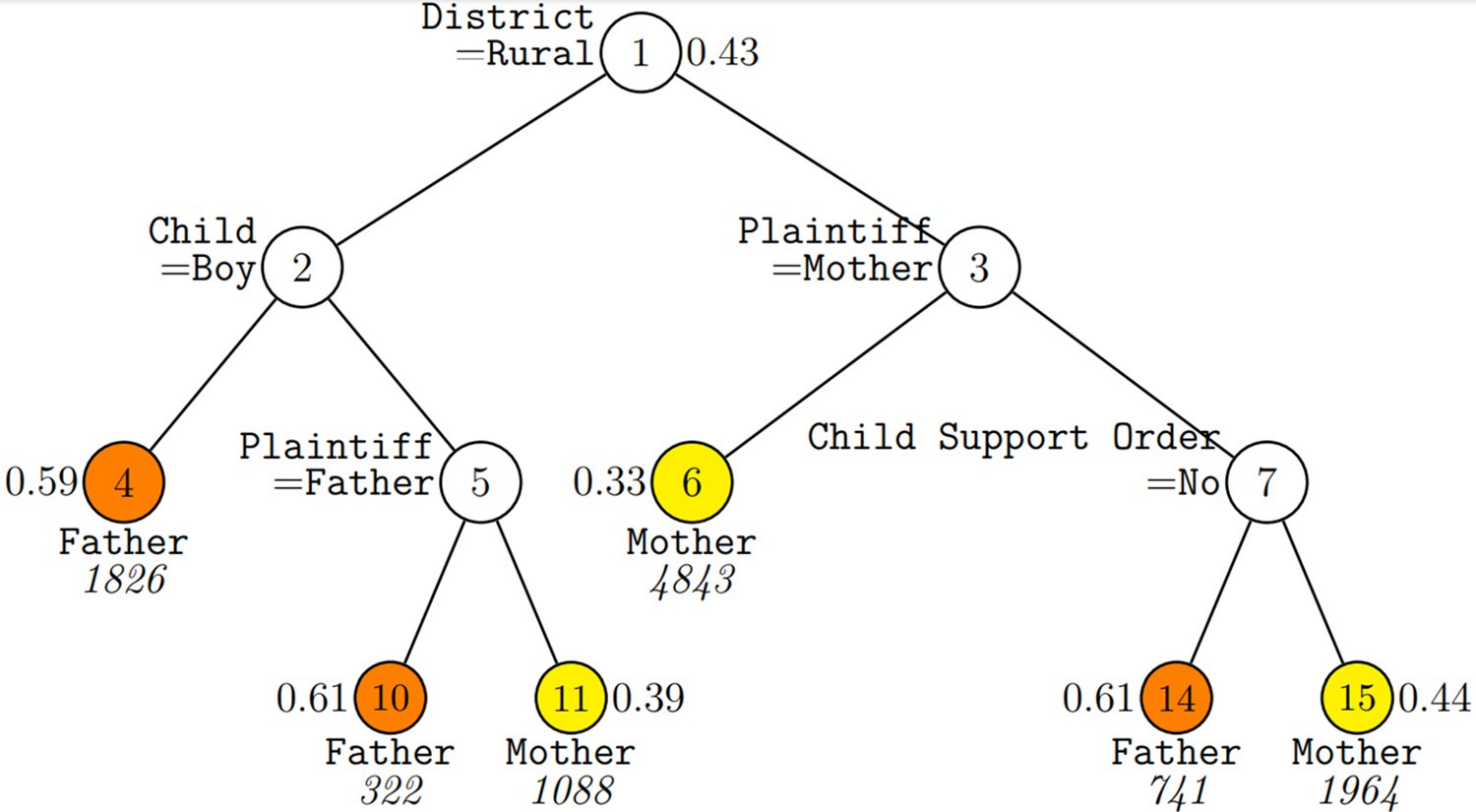
Decision tree result of case outcome vs. all covariates. GUIDE v.41.2 1.000-SE classification tree for predicting which parent the custody is awarded to using estimated priors and unit misclassification costs. At each split, an observation goes to the left branch if and only if the condition is satisfied. Predicted ruling (father or mother) and sample sizes (in italics) are shown below terminal nodes; proportion of cases in which the custody is awarded to father is shown besides the nodes.

Fig 1 illustrates the different gender outcomes in child custody disputes in urban and rural legal settings, highlighting how the dynamics of each setting affect case resolutions. In urban courts, the role of the litigant (plaintiff or defendant) and the presence of child support disputes are key factors. Mothers who initiate litigation (plaintiffs) have a significant advantage, achieving a 67% custody win rate in 4,843 cases. Conversely, mothers who defend, especially in cases involving child support orders or disputes, still maintain a favorable position with a 56%-win rate. On the other hand, fathers in urban settings generally fare less well, but find increased success as plaintiffs in situations without child support disputes, with a success rate of 61%.

Rural court findings further underscore the critical impact of the child’s gender on custody outcomes and illustrate pronounced gender biases. Rural fathers are more likely to be awarded custody when the child is male, as evidenced by a 59% success rate in 1,826 cases, reflecting deep-seated societal preferences for male guardianship, especially of sons. In addition, rural fathers maintain a significant advantage in disputes over daughters, winning custody in 61% of cases where they are the plaintiff. Conversely, rural mothers primarily succeed in gaining custody when they actively pursue legal action for daughters, with a success rate of 61% in 1,088 cases. This trend underscores traditional caregiving roles while highlighting persistent gender biases, particularly related to the child’s gender, across settings.

It also suggests that court decisions are not consistent with the assumption that children should be placed with parents of the same gender, contrary to claims in [55, 56]. This pattern is particularly absent in urban courts. It indicates that assumptions regarding children’s better alignment with same-gender parents lack substantial influence in judicial decisions.

To focus specifically on disputes over the custody of boys in rural courts, we conducted an additional analysis of judicial decisions. According to the data in Table 4, judges are more likely to apply simplified procedures when mothers are the plaintiffs seeking custody. In contrast, regular procedures are more frequently used when fathers initiate custody claims. This trend suggests that cases brought by mothers generally present less conflict, with clearer facts and stronger evidence. On the other hand, fathers pursuing custody tend to face more challenging circumstances, requiring thorough evaluations and careful consideration by the courts.

**Table 4:** Case outcome on boys’ custody by the type of procedures in rural courts.

**Cases in which the plaintiff has asserted custody**		
	**Mother**	**Father**
**Regular**	169	104
**Simplified**	226	81
**P-value**	0.002524	
**Cases awarding custody to defendant when acting as plaintiff**		
	**Mother**	**Father**
**Regular**	315	76
**Simplified**	453	69
**P-value**	0.01096	

Table 4 also shows that when fathers initiate custody disputes and the boy is awarded to the mother, regular procedures are typically followed. Conversely, when mothers initiate and fathers are granted custody, simplified procedures are more commonly used. This pattern may reflect the significant interest fathers have in maintaining custody of sons, highlighting the cultural and familial importance of male offspring in paternal families, as noted by Han [36], Chen and Zhang [24], and Hu and Shen [38].

This subtly suggests that judges’ decisions may be influenced in rural areas where patriarchal values, male-centric norms, and a cultural preference for male children are more pronounced. In such contexts, fathers may become "difficult" defendants and may use aggressive strategies, including verbal threats and physical intimidation, to tip the legal balance in their favor, as noted by Wang [61] and He [35]. These actions may further exacerbate the socioeconomic disadvantages women face [62]. As a result, the legal process in these areas may strengthen paternal rights, reflecting and possibly reinforcing traditional notions of fatherhood prevalent in rural societies.

## 6. Conclusion

This study enriches the extensive literature on child custody disputes by examining the unique context of Chinese courts through a comparative lens. It highlights how the legal system under which Chinese courts operate differs and how the outcomes of cases in these courts compare with those in other legal systems, highlighting both similarities and differences.

On the one aspect, mothers are more likely to be plaintiffs in custody disputes and are proactive in asserting their custody rights, winning custody in 57.81% of cases. More specifically, mothers are significantly more likely to win custody in cases involving daughters, with a 28.14% advantage. In cases involving sons, this advantage drops to 5.28%.

These findings suggest a maternal preference in custody cases. But the likelihood of mothers winning custody in China’s courts is lower compared to the overwhelming advantage observed in Western, European, and other developed societies. In addition, when comparing the case outcomes with the overall parenting arrangements in Chinese society, where approximately 70% of children in single-parent families live with their mothers [66, 67], it suggests that the poorer outcomes for mothers in court compared to negotiation may indicate potential gender bias within the judicial system. Alternatively, this discrepancy might be because fathers with a strong interest or a strong case are unable to persuade mothers to relinquish custody and are thus forced to resort to the courts. Our data, which only include custody disputes resolved through litigation and not through informal procedures such as mediation or negotiation, cannot definitively support one or the other.

On the other hand, the study shows a stark contrast between urban and rural court outcomes. In urban courts, mothers have a clear advantage. In rural courts, mothers are at a disadvantage, winning custody only 47.18% of the time, despite the nearly identical level of effort by mothers in both settings to secure custody. Fathers are particularly assertive in seeking custody of their sons, and mothers are less likely to be awarded custody of their sons in these settings.

This urban-rural disparity raises concerns about the difficulties and barriers women face in rural courts. The observed gendered outcomes may be related to a complex interaction of several factors, such as economic and cultural differences between urban and rural areas. However, by showing that judges in different social contexts make different decisions in similar cases, we raise critical questions about the potential gender bias inherent in the court processes and systems themselves, which is particularly evident and entrenched in rural courts. These questions, supported by previous literature and theoretical perspectives, include the courts’ commitment to upholding women’s rights in marriage and custody disputes, achieving substantive gender equality, and adhering to the principle of serving the best interests of the child. More broadly, it questions whether courts can provide a balanced and fair playing field for all parties involved.

This study serves as a valuable resource for those interested in understanding and addressing gender inequality at its core. It sheds light on the specific challenges women face in custody cases and calls for broader societal and policy changes to support women and combat gender discrimination in all its forms.

## References

[1] Kline Pruett M, DiFonzo JH. Closing the gap: Research, policy, practice, and shared parenting. Fam. Ct. Rev. 2014; 52:152.

[2] Zhang C. Are children from divorced single-parent families disadvantaged? New evidence from the China family panel studies. Chinese Sociological Review. 2020 Jan 1;52(1):84–114.

[3] Palmer M. Transforming family law in post-Deng China: marriage, divorce, and reproduction. China Q. 2007;191: 675–695.

[4] Li H, Xiyuan W, Kaibo Z. An analysis of protections of the rights and interests of children in divorce: divorce cases from economically diverse parts of Jilin Province. J China Womens Univ. 2016;3: 14–22.

[5] Ma L, Rizzi E, Turunen J. Childlessness, sex composition of children, and divorce risks in China. Demographic Research. 2019 Jul 1; 41:753–80.

[6] Xu Q, Yu J, Qiu Z. The impact of children on divorce risk. The Journal of Chinese Sociology. 2015 Dec; 2:1–20.

[7] Zhang J. Snatching and hiding children. Blue book. Available from: https://view.inews.qq.com/k/20230214A089F800?tbkt=H&uid=&refer=wx_hot; 2020.

[8] Zermatten J. The best interests of the child principle: literal analysis and function. The International Journal of Children’s Rights. 2010 Jan 1;18(4):483–99.

[9] Cancian M, Meyer DR. Who gets custody? Demography. 1998;35: 147–157. 9622778

[10] Antokolskaia M. Grounds for divorce and maintenance between former spouses–Russia. University of Utrecht. Prepared for the Commission on European Family Law. Available from: http://ceflonline.net/wp-content/uploads/Russia-Divorce.pdf; 2002.

[11] Australian Institute of Family Studies. Parenting arrangements after separation, EVIDENCE SUMMARY. Available from: https://aifs.gov.au/sites/default/files/publication-documents/1910_parenting_arrangements_after_separation.pdf; 2019.

[12] Vezzetti V. European children and the divorce of their parents: A question of right to health. Contribution to Day general discussion: Digital Media and Children’s rights. Office of High Commissioner for Human Rights. 2014 Sep;12.

[13] McCauley MJ. Divorce and the welfare of the child in Japan. Pac Rim Law Policy J. 2011;20: 589–606.

[14] Mellpy S. Child custody orders: fact or fiction. Paper delivered at Hong Kong Family Law Association/Hong Kong Univ Child Law Conference; 2012.

[15] Neely R. The primary caretaker parent rule: child custody and the dynamics of greed. Yale Law Policy Rev. 1984;3: 168.

[16] Bahr SJ, Howe J. Mann D., morrill Meggin, and Bahr, Matthew S. Fam Law Q. 1994. Trends in Child Custody Awards: Has the Removal of Maternal Preference made a Difference;28: 247.

[17] Leving JM, Dachman KA. Fathers’ rights: hard hitting and fair advice for every father involved in a custody dispute. New York: Basic Books; 1997.

[18] Polikoff ND. Why are Mothers Losing: A brief analysis of criteria used in child custody determinations. Women’s Rights Law Report. 1983;7: 235–243.

[19] Albertson Fineman M. 1991. The illusion of Equality: the rhetoric and reality of divorce reform. Chicago: University of Chicago Press.

[20] Chesler P. Mothers on trial: the Battle for children and custody (revised). Chicago, IL: Lawrence Hill Books; 2011.

[21] Xi Jinping. 2020. General Assembly high-level meeting on the 25th anniversary of the Fourth World Conference on Women. United Nations.

[22] Supreme People’s Court. (2022). Report on the Work of Family Trials in National Courts since the 18th National Congress of the Communist Party of China. Available from: https://www.court.gov.cn/fabu-xiangqing-376111.html

[23] Santos G, Harrell S, editors. Transforming patriarchy: Chinese families in the twenty-first century. University of Washington Press; 2016 Nov 1.

[24] Chen W, Zhang Q. Judicial practice of child support in divorce litigation and its suggestions for improvement: A survey of divorce cases concluded in a County Court from 2011 to 2013. Hebei Jur. 2015;33: 21. (in Chinese).

[25] Zhao L, Ding Y. Problems and countermeasures in divorce cases involving the attribution of custody of minor children: A sample of four years (2011−2014) of divorce dispute judgements from six basic courts in Nanjing. J China. Coll: Women’s Press. 2016;1: 11.

[26] Michelson E. Possession is nine-tenths of the law: why wife-beaters are awarded child custody in China’s divorce courts. SSRN Journal. 2020;15: 126–159.

[27] Michelson E. Decoupling: gender injustice in China’s divorce courts (Cambridge Studies in Law and Society). Cambridge: Cambridge University Press; 2022.

[28] Chen YJ, Chen Z, He S. Social norms and household savings rates in China. Review of Finance. 2019 Sep 1;23(5):961–91.

[29] Zhang C. Family support or social support? The role of clan culture. Journal of Population Economics. 2019 Apr; 32:529–49.

[30] Das Gupta M, Shuzhuo Li. Gender bias in China, the Republic of Korea, and India 1920–90–effects of War, famine, and fertility decline. Policy Research Working Paper Series 2140. Washington, DC: The World Bank; 1999.

[31] Woo MYK. Shaping citizenship: Chinese family law and women. SSRN Journal. 2003;15: 99.

[32] He X, Ng K. Pragmatic discourse and gender inequality in China. Law Soc Rev. 2013;47: 279–310.

[33] He X, Ng K. In the name of harmony: the erasure of domestic violence in China’s judicial mediation. Int J Law Policy Fam. 2013;26: 97–115.

[34] Li K. What He did was lawful: divorce litigation and gender inequality in China. Law Policy. 2015;37: 153–179.

[35] He X. “No Malicious Incidents”: the concern for stability in China’s divorce law practice. Soc Leg Stud. 2017;26: 467–489.

[36] Han P. A legal sociological analysis of the adjudication process of grassroots courts: A cut to the child support issue in divorce cases. Nanjing Univ. Law Rev. 2014;1: 35.

[37] Liebman BL, Roberts ME, Stern RE, Wang AZ. Mass Digitization of Chinese Court Decisions. J Law Courts. 2020;8: 177–201.

[38] Hu M, Shen X. An empirical investigation study on the legal protection of children’s rights and interests in divorce. Proceedings of a survey of divorce cases concluded in three basic courts in Hainan province. Humanit Soc Sci. *Journal of Hainan University*. 2016;10: 34.3.

[39] Maccoby E, Mnookin RH. Dividing the child: social and legal dilemmas of custody. Cambridge, MA: Harvard University Press; 1992.

[40] Brinig MF, Allen DW. These boots are made for walking: why most divorce filers are women. Am Law Econ Rev. 2000;2: 126–169.

[41] Hamer J, Marchioro K. Becoming custodial fathers: exploring parenting among low-income and working-class African-American fathers. J Marriage Fam. 2002;64: 116–129.

[42] Arditti JA, Madden-Derdich DA. Noncustodial mothers: developing strategies of support. Fam Relat. 1993;42: 305–314.

[43] Herreras C. Noncustodial mothers following divorce. Marriage Fam Rev. 1994;20: 233–255.

[44] Seltzer JA. Legal custody arrangements and children’s economic welfare. Am J Sociol. 1991;96: 895–929.

[45] He X. Let Men Come out Ahead: Property Division in China’s Divorce Cases. J. Comp. L. 2020; 15:204.

[46] Hu Y, Scott J. Family and gender values in China: Generational, geographic, and gender differences. Journal of Family Issues. 2016 Jun;37(9):1267–93.

[47] Yang XY, Wu N, Hou J. Gender-health disparities: exploring the counterbalancing mechanisms of labor disadvantage and health behaviors in rural China. China Population and Development Studies. 2022 Jun;6(2):186–205.

[48] Judd ER. No change for thirty years: The renewed question of women’s land rights in rural China. Development and Change. 2007 Jul;38(4):689–710.

[49] Blau FD, Kahn LM, Brummund P, Cook J, Larson-Koester M. Is there still son preference in the United States? Journal of Population Economics. 2020 Jul; 33:709–50.

[50] Song Y, Gao J. Do fathers have son preference in the United States? Evidence from paternal subjective well-being. Review of Economics of the Household. 2023 Jan 2:1–35.

[51] Murphy R, Tao R, Lu X. Son preference in rural China: Patrilineal families and socioeconomic change. Population and development review. 2011 Dec;37(4):665–90. doi: 10.1111/j.1728-4457.2011.00452.x 22319769

[52] Lei L, Pals H. Son preference in China: Why is it stronger in rural areas? Population review. 2011;50(2).

[53] Raday F. Gender equality and women’s rights in the context of child custody and maintenance: an international and comparative analysis. UN women discussion paper series No. 30. Women. New York: United Nations. 2019.

[54] Chambers DL. Rethinking the substantive rules for custody disputes in divorce. Mich Law Rev. 1984;83: 477–569.

[55] Shanghai Second Intermediate People’s Court. White paper on trials of divorce cases involving minor children in the second intermediate juvenile division 2016–2017.

[56] Xiangzhou District People’s Court. Zhuhai City, Guangdong Province. Research on the Protection of Children’s Rights and Interests in Family Trials. Available from: http://www.zhxzcourt.gov.cn/index.php?do=court&ac=info&cid=3290; 2017.

[57] Zhu X. Guidelines for assessing the principle of the best interests of minors in custody disputes. Legal science. J Northwest Univ Pol Sci Law. 2020;38: 14.

[58] Supreme People’s Court. National court judicial statistics. Available from: http://gongbao.court.gov.cn/Details/a6c42e26948d3545aea5419fa2beaa.html; 2021.

[59] Supreme People’s Court. Opinions on Handling Child Support Issues in Divorce Cases. [1993] No. 30.

[60] Priest GL, Klein B. L. and Klein. The selection of disputes for litigation. J Leg Stud. 1984;13: 1–55.

[61] Wang J. To divorce or not to divorce: A critical discourse analysis of court ordered divorce mediation in China. Int J Law Policy Fam. 2013;27: 74–96.

[62] Wang J. Hanyin County People’s court, A few thoughts on the high divorce rate in rural areas. Available from: http://sxhyfy.sxfywcourt.gov.cn/article/detail/2013/06/id/4427334.shtml; 2013.

[63] Yu X, Sun Z. The company they keep: when and why Chinese judges engage in collegiality. J Empirical Leg Stud. 2022;19: 936–1002.

[64] Chen M, Rizzi EL, Yip PS. Divorce trends in China across time and space: an update. Asian Population Studies. 2021 May 4;17(2):121–47.

[65] Loh WY. Regression tress with unbiased variable selection and interaction detection. Statistica sinica. 2002 Apr 1:361–86.

[66] National Health and Family Planning Commission of the People’s Republic of China. China Family Development Report 2014. Beijing: China Population Publishing House; 2014. (in Chinese).

[67] Lin WJ, Zhao YH. Does son preference reduce female welfare? Divorce and parenting stress. Economics Quarterly 2015; 1:135–58. (in Chinese).

